# Comparison of proteomic profiles of serum, plasma, and modified media supplements used for cell culture and expansion

**DOI:** 10.1186/1479-5876-4-40

**Published:** 2006-10-04

**Authors:** Saleh Ayache, Monica C Panelli, Karen M Byrne, Stefanie Slezak, Susan F Leitman, Francesco M Marincola, David F Stroncek

**Affiliations:** 1 Department of Transfusion Medicine, Warren G. Magnuson Clinical Center National Institutes of Health, Bethesda, Maryland, USA

## Abstract

**Background:**

The culture and expansion of human cells for clinical use requires the presence of human serum or plasma in culture media. Although these supplements have been extensively characterized in their chemical composition, only recently it has been possible to provide by high throughput protein analysis, a comprehensive profile of the soluble factors contributing to cell survival. This study analyzed and compared the presence of 100 proteins including chemokines, cytokines and soluble factors in six different types of media supplements: serum, plasma, recalcified plasma, heat inactivated serum, heat inactivated plasma and heat inactivated recalcified plasma.

**Methods:**

Serum, plasma, recalcified plasma, and heat inactivated supplements were prepared from ten healthy subjects. The levels of 100 soluble factors were measured in each sample using a multiplexed ELISA assay and compared by Eisen hierarchical clustering analysis.

**Results:**

A comparison of serum and plasma levels of soluble factors found that 2 were greater in plasma but 18 factors were greater in serum including 11 chemokines. The levels of only four factors differed between recalcified plasma and plasma. Heat inactivation had the greatest effect on soluble factors. Supervised Eisen hierarchical clustering indicated that the differences between heat inactivated supplements and those that were not were greater than the differences within these two groups. The levels of 36 factors differed between heat inactivated plasma and plasma. Thirty one of these factors had a lower concentration in heat inactivated plasma including 12 chemokines, 4 growth factors, 4 matrix metalloproteases, and 3 adhesion molecules. Heat inactivated decalcified plasma is often used in place of heat inactivated serum and the levels of 19 soluble factors differed between these two supplements.

**Conclusion:**

Our report provides a comprehensive protein profile of serum, plasma recalcified plasma, and heat inactivated supplements. This profile represents a qualitative and quantitative database that can aid in the selection of the appropriate blood derived supplement for human cell cultures with special requirements.

## Background

There is a growing interest in the in vitro generation of large numbers and varieties of cell types for clinical applications including dendritic cells (DCs) to improve the effectiveness of immune therapies [1-4], cytotoxic T cell therapy of cancer [5], EBV induced lymphoma [6], and CMV infection [7,8], and to expand hematopoietic stem cells to provide greater quantities of transplantable cells [9,10]. The culture and expansion of cells usually involves the addition of cytokines and/or growth factors. In addition, serum or plasma is often added to the culture media [1,3,6-8].

Although fetal calf serum has often been the standard media supplement for in vitro research studies, fetal calf serum is not suitable for the production of cells for clinical use. The use of bovine serum exposes patients to the potential risk of transmission of pathogens such as Creutzfeldt-Jakob disease (CJD) [11] and the development of a humoral or cellular response to bovine proteins [4]. In spite of these issues, some clinical protocols use fetal calf serum [3,6], but often human serum is used in place of fetal calf serum [1,7,8]. In some cases human plasma [1] or no media supplement is used [2,4,9,10].

While human serum is likely the best substitute for fetal calf serum, human plasma is more readily available than serum. Plasma is regularly produced by blood centers following good manufacturing practices from donors screened and tested for transfusion transmitted diseases. The plasma is stored frozen and is used for transfusion. In contrast, serum is not used for transfusion and is not routinely manufactured by blood centers.

The use of human plasma rather than serum in cell culture, however, presents some problems. Citrate, a calcium chelator, is the standard plasma anticoagulant used in the process of plasma collection. When plasma is added to culture media which usually contains calcium, fibrin or clots may form. This can be prevented by adding thrombin and calcium to plasma to induce clot formation and defibrination. Recalcified plasma is used in place of fetal calf serum in some clinical protocols.

When serum or plasma is used as a culture media supplement, it is often modified by heat inactivation which involves heating at 56°C for 1 hour to inactivate complement components and prevent the occurrence of complement mediated lysis in cell cultures [1]. However, heating has a large spectrum of denaturing effects and hence affects other serum and plasma factors.

Although serum, plasma, recalcified plasma, and heat inactivated serum and plasma are used to supplement culture media little is known about the differences in the composition of these supplements. The goal of this study was to determine if differences in the levels of soluble factors among these media supplements might contribute to the differences in their efficacy. Results from this study may assist in determining which plasma or plasma-derived components would be more appropriate for human cell cultures with special requirements.

## Methods

### Study design

A total of 450 mL of whole blood was collected from each of 10 healthy research donors. The blood was collected in two aliquots: one of 375 mL and the other 125 mL. The larger aliquot was collected in CP2D (citrate-phosphate-2dextrose) anticoagulant and was used to prepare plasma. One third of plasma prepared from the CP2D blood was processed to make recalcified plasma, one third to make heat inactivated plasma, and one third was not further processed. The 125 mL aliquot of blood was collected in a bag without anticoagulant and was used to make serum. One and one-half mL aliquots from all components were frozen at -80°C. Aliquots from each of the 10 subjects were tested for 100 soluble factors using a multiplex ELISA. In the first part of the study serum, plasma, recalified plasma and heat inactived plasma were tested. In the second part of the study samples of serum, plasma, and recalcified plasma stored at -80°C were thawed, heat inactivated and tested.

### Blood collection

Whole blood was collected using a specially assembled set of blood collection bags consisting of a dry bag from a Pall collection bag system (Leukotrap WB system, CP2D/AS-3 double blood bag unit, Pall Corporation, East Hills, NY 11548, USA) and Baxter, 150 mL transfer bag (Baxter Health Corporation, Fenwal division, Deerfield, IL 60015, USA). The blood collection bags were assembled using a sterile connecting device (SCD 312, Terumo Medical Corp, Elkon, MD). The seven bag set included a phlebotomy needle tubing which was connected to a Y anastomoses tubing with one side connected to a collecting bag labeled (A), which contained 55 mL of CP2D as anticoagulant. Bag A was connected to a dry satellite bag labeled B, which in turn was connected to three 150 mL transfer bags labeled C, D, and E. The second end of Y blood withdrawal tubing was connected to a dry CLX satellite bag (Pall) labeled F, which was connected to second dry bag labeled G.

After 375 mL of whole blood was collected into the first primary bag (A), the tubing leading to bag (F) was then opened and 125 ml of blood was collected into this bag (F) which was placed on ice during phlebotomy. Bags A and F were separated at the end of the collection and the blood in bag A was used to prepare plasma and plasma products, while blood in bag F was used to prepare serum.

### Plasma and serum preparation and storage

To prepare plasma the whole blood in bag A was centrifuged at 4000 rpm for 6.5 minutes (RC3C, Sorval Instruments, Newton, CT). Plasma was expressed into bag B which was then divided equally between the three satellite bags (C, D, and E) with a final volume of 60 to 75 mL in each bag. Plasma bag E was not processed further.

To prepare recalcified plasma calcium chloride (CaCl_2_) and thrombin were added to bag C and the plasma was incubated at 37°C for one hour and then stored at 4°C overnight. The bag was then centrifuged at 4000 rpm for 6.5 minutes at room temperature and supernatant, recalcified plasma was expressed into a satellite bag.

Heat inactivated plasma was prepared by incubating plasma in bag D at 56°C for one hour. The bag was then centrifuged at 4000 rpm for 6.5 minutes and the expressed supernatant, heat inactivated plasma, was transferred into satellite bag.

To prepare serum, the whole blood in bag F was stored on ice for one hour. It was then centrifuged at 4000 rpm for 6.5 minutes and the supernatant was transferred into bag G.

Immediately after the preparation of each component was complete, 1.5 mL aliquots were placed into cryovials (1.8 mL Nunc) and snap frozen on dry ice and ethanol. After freezing, all samples were then transferred to -80°C for storage.

### Analysis of plasma and serum proteins

The levels of 100 soluble factors were assessed on an ELISA-based platform (Pierce Search Light Proteome Arrays, Boston, MA) consisting of multiplexed assays that measured up to 16 proteins per well in standard 96 well plates[12]. The arrays were produced by spotting 2 × 2, 3 × 3, or 4 × 4 patterns of different monoclonal antibodies into each well of a 96-well plate. Following a typical sandwich ELISA procedure, signal was generated using a chemiluminescent substrate. The light produced at each spot in the array was captured by imaging the entire plate with a commercially available cooled CCD camera. The data was reduced using image analysis software that calculated exact values (pg/mL) based on standard curves. The 100 factors included: interferon alpha (IFN-α), IFN gamma (IFN-γ), interleukin 1 alpha (IL-1α), IL-1β, IL-1Rα, IL-2, IL-3, IL-4, IL-5, IL-6, IL-7, IL-8, IL-9, IL-10, IL-11, IL-12p40, IL-12p70, IL-13, IL-15, IL-16, IL-18, tumor necrosis factor alpha (TNF-α), epithelial cell-derived neutrophil activating protein 78 (ENA-78), eotaxin/CCL11, eotaxin II/CCL24, exodus II, growth related oncogene alpha (GRO-α/CXCL1), inducible 309 (I-309/CCL1), interferon inducible protein 10 (IP-10/CXCL10), interferon-inducible T-cell alpha chemoattractant (ITAC/CXCL11), lymphotactin (LTN), monocyte chemoattractant protein 1(MCP-1/CCL2), MCP-2/CCL8, MCP-3/CCL7, MCP-4/CCL13, macrophage-derived chemokine (MDC/CCL22), monokines induced by interferon (MIG/CXCL9), macrophage inflammatory protein 1α (MIP-1α/CCL3), MIP-1β/CCL4, MIP-3α/CCL20, MIP-3β/CCL19, myeloid progenitor inhibitory factor (MPIF-1), neutrophil chemoattractant peptide 2 (NAP-2/CXCL7), stromal-derived factor 1β (SDF-1β/CXCL12), regulated on activation normal T cell-expressed and secreted (RANTES/CXCL5), thymus and activation regulated chemokine (TARC/CCL17), granulocyte colony-stimulating factor (G-CSF), granulocyte macrophage-colony stimulating factor (GM-CSF), angiogenesis factors, angiopoietin-2 (ANG-2), fibroblast growth factor-α (FGF-α) fibroblast growth factor basic (FGF-b), keratinocyte growth factor (KGF), hepatocyte growth factor (HGF), heparin binding epidermal growth factor (HB-EGF), platelet derived growth factor BB (PDGF-BB), vascular endothelial growth factor (VEGF), human growth hormone (HGH), TGF-α, matrix metalloproteinase 1 (MMP-1), MMP-2, MMP-3, MMP-8, MMP-9, MMP-10, MMP-13, tissue inhibitor of metalloprotease 1 (TIMP-1), TIMP-2, brain-derived neurotrophic factor (BDNF), nerve growth factor-beta (β-NGF), ciliary neurotrophic factor (CNTF), neurotrophin-3 (NT3), leukemia inhibitory factor (LIF), serum amyloid A (SAA), pregnancy associated plasma protein-A (PAPP-A), amphiregulin (AR), leptin, adiponectin (ACRP-30) adipocytes secreted proteins, Apo-A1, ApoB-100, soluble CD14 (sCD14), CD40 ligand (CD40L), C reactive protein (CRP), myeloperoxidase (MPO), fibrinogen, osteoprotegrin (OPG), osteopontin (OPN), plasminogen activator inhibitor-1 Active (PAI-1), PAI-1Total, aminoterminal proBNP (NT-proBNP), receptor activator of nuclear factor-kappa B (RANK), receptor activator of nuclear factor-kappa B ligand antibodies (RANK-L), vascular cell adhesion molecule (VCAM-1), intracellular adhesion molecule (ICAM-1), L-Selectin, E-Selectin, TNF receptor-1 and 2 (TNFR1, TNFR2), IL-1 receptor (IL-2R), and IL-6R.

### Statistical analysis

Paired two tailed *t-*test of natural log transformed data was used to compare the plasma and serum soluble factor levels. Differences were considered significant at a cut off p-value of ≤ 0.01. Relatedness of cytokine expression patterns in different types of plasma and serum was tested by applying unsupervised Eisen's hierarchical clustering methods [13] to the data set encompassing the soluble factors across all samples. Unsupervised clustering involved the sorting of both the soluble factors and the samples. In contrast, for unsupervised clustering the factors were sorted, but not the samples. Twenty of the 100 factors were excluded from the analysis since their levels were under the assay's limits of detection. Those factors were: TGF-β, IL-1β, IL-4, IL-5, IL-10, IL-12p70, IL-13, IL-15, IL-17, LIF, NT-proBNP, RANK, RANK-L, PAPPA, MMP-8, B-NGF, VEGF, HB-EGF, IL-3, B-NGF, and FGF-α.

## Results

### Proteomic profiles of serum, plasma, recalcified plasma and heat inactivated plasma

**Figure 1:**
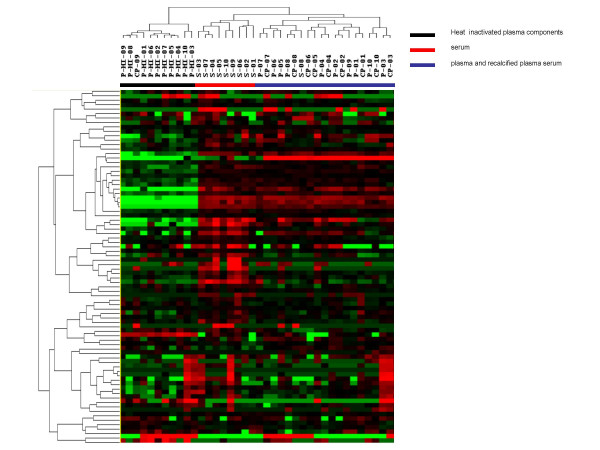
Unsupervised Hierarchical clustering (Eisen Kendall's) of 80 soluble factor levels among a total of 40 samples including 10 serum (S), 10 plasma (P), 10 recalcified plasma (CP), and 10 heat inactivated plasma (P-HI) samples prepared from whole blood from 10 healthy subjects (01 through 10). The levels of the soluble factors were measured by multiplexed ELISA.

Analysis of the levels of the 80 factors using unsupervised hierarchical clustering among serum, plasma, recalcified plasma components and heat inactivated plasma prepared from 10 subjects yielded three groups of factors. The first group was made up predominantly of heat inactivated plasma components (Figure 1, black bar), the second of serum components (Figure 1, red bar), and the third of plasma and recalcified plasma components (Figure 1, blue bar). The heat inactivated group contained 11 samples: all 10 heat inactivated plasma samples (P-HI-01, P-HI-02, P-HI-03, P-HI-04, P-HI-05, P-HI-06, P-HI-07, P-HI-08, P-HI-09, and P-HI-10) and one recalcified plasma sample (CP-9). The serum group contained 9 serum samples (S-01, S-02, S-03, S-04, S-05, S-06, S-07, S-09, and S-10). The plasma and recalcified plasma group was constituted by 20 samples: 10 plasma (P-01, P-02, P-03, P-04, P-05, P-06, P-07, P-08, P-09, and P-10), 9 recalcified plasma (CP-01, CP-02, CP-03, CP-04, CP-05, CP-06, CP-07, CP-08, and CP-10) and one serum (S-08) samples. The mean levels of all 80 factors are shown in Additional file 1.

**Figure 2:**
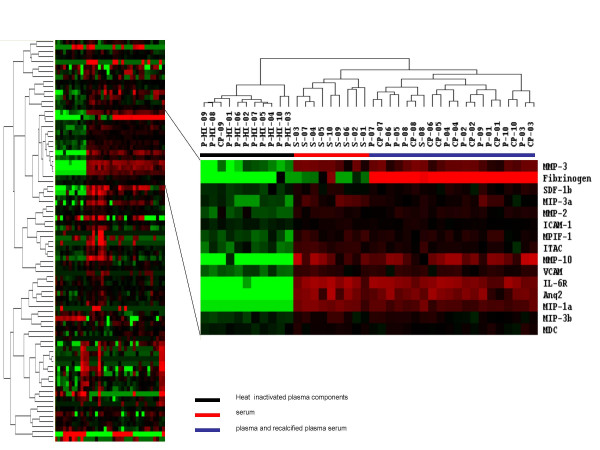
Factors that distinguish heat inactivated plasma from plasma, serum, and recalcified plasma. Hierarchical clustering analysis was applied to 80 soluble factors measured in a total of 40 serum, plasma, and plasma derived components (as per Figure 1). The ten heat inactivated plasma components separated into a single group with one recalcified plasma component. A cluster of 15 soluble factors best distinguished the heat inactivated group from the other 29 components.

The most prominent dissimilarity among the 40 samples was a cluster of 15 factors whose levels were markedly lower in the 10 heat inactivated plasma than in the other samples (Figure 2). These factors were fibrinogen, MIP-1α, MIP-3α, MIP-3β, MDC, MMP-2, MMP-3, MMP-10, ICAM-1, VCAM, SDF-1β, ITAC, MPIF-1, IL-6R, and Ang2.

These results show that plasma and recalcified plasma are very similar in regards to the factors analyzed. However, serum and plasma are quite different as previously reported by our group [14] and heat inactivation affected the levels of several factors.

### Comparison of serum and plasma

Previous studies have found that the levels of several factors differed between serum prepared from blood collected in BD vacutainer serum collection tubes and plasma prepared from blood collected in vacutainers with EDTA [14]. A comparison of the soluble factors in the 10 plasma samples prepared in this study from citrated blood collected in bags and the 10 serum samples collected in bags revealed that the levels of 20 factors differed between serum and plasma (Table 1). The levels of fibrinogen and OPN were greater in plasma and levels of 18 factors were greater in serum. Among the other 18 factors whose levels were greater in serum than in plasma were 11 chemokines (MCP-2, MCP-3, MIP-1α, MIP-1β, MIP-3α, NAP-2, ITAC, lymphotactin, eotaxin 2, exodus 2, and RANTES) and 4 factors normally found in platelets NAP-2, RANTES, PDGF-BB, and PAI-1 total.

**Table 1:** Factors Whose Levels Differed Between Serum and Plasma Components

	**Factor Levels**			
			
**Factor**	**Serum**	**Plasma**	**Fold Difference**	p*
**Factor whose levels were greater in plasma**				
Fibrinogen (microg/mL)	3.13 ± 7.21	4,812 ± 1,780	24,500± 20,700	2.54E-07
OPN (ng/mL)	1.82 ± 1.18	4.12 ± 1.48	2.7 ± 1.0	5.61E-05

**Factors whose levels were greater in serum**				
TNFR1 (pg/mL)	582 ± 139	422 ± 103	1.39 ± 0.21	7.46E-05
MIP-1α (pg/mL)	96.2 ± 22.0	56.4 ± 8.8	1.71 ± 0.35	0.00014
ITAC (pg/mL)	78.4 ± 15.8	51.0 ± 7.3	1.55 ± 0.27	0.00014
NAP-2 (microg/mL)	4.79 ± 7.16	0.183 ± 0.321	62.48 ± 96.9	0.00019
Lymphotactin (pg/mL)	125 ± 104	103 ± 96	1.30 ± 0.20	0.00056
MIP-1β (pg/mL)	98.5 ± 64.0	74.3 ± 58.6	1.39 ± 0.31	0.0012
MCP-2 (pg/mL)	47.1 ± 11.3	31.1 ± 10.2	1.62 ± 0.50	0.0013
TIMP-1 (ng/mL)	106.3 ± 25.167	71.8 ± 8.36	1.50 ± 0.39	0.0016
PAI-1 total (pg/mL)	4,177 ± 3,170	929 ± 746	9.19 ± 15.21	0.0023
Eotaxin 2 (pg/mL)	1,976 ± 1,366	988 ± 576	1.93 ± 0.84	0.0028
MIP-3α (pg/mL)	17.1 ± 7.0	11.8 ± 6.3	1.53 ± 0.47	0.0030
Exodus 2 (pg/mL)	57.3 ± 16.5	48.7 ± 10.6	1.17 ± 0.15	0.0048
RANTES (ng/mL)	22.0 ± 20.2	5.77 ± 2.22	4.67 ± 5.19	0.0051
MMP-9 (ng/mL)	32.1 ± 23.5	11.4 ± 4.4	3.38 ± 3.24	0.0064
PDGF-BB (pg/mL)	214 ± 225	32.7 ± 19.8	9.97 ± 12.29	0.0072
ACRP-30 (microg/mL)	15.52 ± 14.83	11.26 ± 11.33	1.50 ± 0.57	0.0076
APO-A1 (microg/mL)	208.2 ± 38.49	173.2 ± 46.95	1.25 ± 0.26	0.0089
MCP-3 (pg/mL)	4.7 ± 3.9	1.5 ± 2.5	3.10 ± 0.41	0.0096

*p values were calculated using Student's paired T tests and natural log transformed data.

Values are mean ± one standard deviation.

### Comparison of factor levels in recalcified plasma, regular plasma and serum

To produce recalcified plasma thrombin and calcium were added to plasma. As expected, the levels of fibrinogen were lower in recalicified plasma than in plasma, (262 ± 38 microg/mL vs 4,810 ± 1,780 microg/L, p = 0.026). The levels of only 4 other factors differed between recalcified plasma and plasma (MDC, NAP-2, IL-16 and IFN-α) (Table 2). The levels of MDC, IL-16, and IFN-α were greater in plasma and the level of NAP-2 was greater in recalcified plasma.

**Table 2:** Factors Whose Levels Differed Between Plasma and Recalcified Plasma Components

	**Factor Levels**			
**Factor**	**Plasma**	**Recalified Plasma**	**Fold Difference**	p*

**Factors whose levels were greater in plasma**				
MDC (pg/mL)	350 ± 71	271 ± 50.2	1.30 ± 0.18	0.00016
IL-16 (pg/mL)	711 ± 573	465 ± 513	2.11 ± 1.29	0.0033
IFN-α (pg/mL)	11.5 ± 11.2	4.0 ± 4.8	2.87 ± 1.13	0.0095

**Factors whose levels were greater in recalcified plasma**				
NAP-2 (ng/mL)	183.8 ± 321.1	263.9 ± 305.9	1.92 ± 0.59	0.00033

*p values were calculated using paired Student's T tests and natural log transformed data.

Values are mean ± one standard deviation.

There were more differences between recalcified plasma and serum than between recalcified plasma and plasma. The levels of 19 factors differed between recalcified plasma and serum (Table 3). Eighteen of the 19 factors were greater in serum and one of the factors was greater in recalcified plasma, OPN. Fourteen of the 19 factors that differed among plasma and serum also differed among recalcified plasma and serum (OPN, MIP-1β, MIP-3α, NAP-2, APO-A1, eotaxin 2, MCP-2, MCP-3, TIMP-1, Lymphotaxin, PDGF-BB, ITAC, PAI-1 total, and TNFR1). The 5 factors that differed among recalcified plasma and serum but not plasma and serum were MDC, MMP-2, L-selectin, APO-B100, and ENA-78.

**Table 3:** Factors Whose Levels Differed Between Recalcified Plasma and Serum

	**Factor Levels**			
**Factor**	**Serum**	**Recalcified Plasma**	**Fold Difference**	p*

**Factor whose levels were greater in recalcified plasma**				
OPN (pg/mL)	1.82 ± 1,18	3.92 ± 1.38	2.6 ± 1.0	5.25E-05

**Factors whose levels were greater in serum**				
MIP-1β (pg/mL)	98.5 ± 64.0	60.2 ± 24.7	1.55 ± 0.34	0.00012
NAP-2 (microg/mL)	4.79 ± 7.16	0.264 ± 0.306	31.3 ± 43.9	0.00054
APO-A1 (microg/mL)	208.2 ± 38.49	173.6 ± 36.09	1.21± 0.15	0.00083
Eotaxin 2 (ng/mL)	1.98 ± 1.37	1.04 ± 0.747	1.89 ± 0.68	0.00094
MCP-2 (pg/mL)	47.1 ± 11.3	27.6 ± 10.5	1.92 ± 0.82	0.0010
TIMP-1 (ng/mL)	106.3 ± 25.2	71.3 ± 13.6	1.53 ± 0.44	0.0013
Lymphotactin (pg/mL)	125 ± 104	76.7 ± 51.0	1.73 ± 0.69	0.0014
MDC (pg/mL)	340 ± 56	271 ± 50	1.28 ± 0.24	0.0023
MMP-2 (ng/mL)	116.9 ± 20.18	105.5 ± 16.9	1.11 ± 0.09	0.0031
L-Selectin (microg/mL)	2.24 ± 0.35	2.00 ± 0.19	1.12 ± 0.09	0.0032
MIP-3α (pg/mL)	17.1 ± 7.0	9.1 ± 3.2	2.08 ± 1.34	0.0032
MCP-3 (pg/mL)	4.7 ± 3.9	0.5 ± 1.5	9.4 ± 1.62	0.0036
PDGF-BB (pg/mL)	213.8 ± 225.5	23.7 ± 22.2	9.2 ± 15.2	0.0036
ITAC (pg/mL)	78.4 ± 15.8	47.9 ± 17.9	1.97 ± 1.16	0.0043
PAI-1 total (ng/mL)	4.18 ± 3.17	1.12 ± 1.03	7.99 ± 11.57	0.0047
APO-B100 (microg/mL)	535.3 ± 213.6	358.9 ± 121.2	1.54 ± 0.48	0.0074
ENA-78 (pg/mL)	1,449 ± 787	718 ± 317	2.48 ± 1.89	0.0082
TNFR1 (pg/mL)	582 ± 139	485 ± 94	1.20 ± 0.21	0.0094

*p values were calculated using paired Student's T test and natural log transformed data.

Values are mean ± one standard deviation.

### Comparison of factor levels between freshly collected plasma and heat inactivated plasma

Heat inactivation was performed on a selected set of supplement samples right after collection to make a preliminary assessment of heat-induced protein levels variation. Plasma was selected as the candidate supplement for testing. A comparison of factor levels between heat inactivated plasma and plasma found that the levels of 36 factors changed with heat inactivation of plasma (Table 4). The levels of 5 factors, MPO, MCP-1, eotaxin 2, MIP-1, PAI-1 total, and TNFR1, were greater in heat inactivated plasma (Table 4). The 31 factors whose levels were greater in plasma than in heat inactivated plasma included several chemokines and growth factors.

**Table 4:** Factors Whose Levels Differed Between Heat Inactivated Plasma and Plasma

	**Factor Levels**			
**Factor**	**Plasma**	**Heat Inactivated Plasma**	**Fold Difference**	p*

**Factors whose levels were greater in heat inactivated plasma**				
MPO (pg/mL)	359 ± 245	2,703 ± 1,171	9.29 ± 4.56	5.42E-07
Eotaxin 2 (pg/mL)	989 ± 576	2,352 ± 1,376	2.57 ± 1.04	0.00012
MCP-1 (pg/mL)	361 ± 97	425 ± 111	1.18 ± 0.10	0.00014
PAI-1 total (pg/mL)	929 ± 745	1,709 ± 183	1.62 ± 0.59	0.0026
TNFR1 (pg/mL)	422 ± 103	499 ± 116	1.19 ± 0.20	0.0093

**Factors whose levels were greater in plasma**				
MIP-1α (pg/mL)	56.4 ± 8.8	0.0 ± 0	NA	2.76E-14
Ang 2 (pg/mL)	212 ± 95.2	0.0 ± 0	NA	8.61E-11
MDC (pg/mL)	350 ± 70.6	219.7 ± 36.3	1.58 ± 0.13	2.16E-08
IL-6R (ng/mL)	13.2 ± 4.45	0.043 ± 0.072	307 ± 563	6.67E-08
SDF-1β (pg/mL)	798 ± 137	336 ± 95.7	2.46 ± 0.47	1.86E-07
MMP-10 (pg/mL)	291 ± 183	3.3 ± 10.3	88.2 ± 10.2	2.03E-07
VCAM (ng/mL)	556 ± 133	113 ± 43.4	5.44 ± 2.23	5.36E-07
Fibrinogen (microg/mL)	4,812 ± 1,779	0.107 ± 0.300	45,029 ± 23,528	1.16E-06
PAI-1active (pg/mL)	4,192 ± 4,600	67.9 ± 89.0	61.7 ± 14.5	2.02E-06
MMP-2 (ng/mL)	114 ± 8.4	34.1 ± 21.4	4.01 ± 1.62	7.45E-06
ICAM-1 (ng/mL)	321 ± 37.9	201 ± 242	1.62 ± 0.3	1.33E-05
CRP (ng/mL)	1,749 ± 1,732	729 ± 921	2.75 ± 0.97	1.99E-05
MPIF-1 (pg/mL)	1,618 ± 368	483 ± 214	4.26 ± 3.18	2.12E-05
PDGF-BB (pg/mL)	32.7 ± 19.8	7.3 ± 17.7	4.48 ± 1.65	4.44E-05
MIP-3β (pg/mL)	88.2 ± 22.4	43.6 ± 11.7	2.11 ± 0.67	4.65E-05
MIP-3α (pg/mL)	11.8 ± 6.3	2.3 ± 2.1	5.13 ± 1.51	6.24E-05
ITAC (pg/mL)	51.0 ± 7.3	17.6 ± 9.9	3.78 ± 1.88	6.84E-05
MMP-3 (ng/mL)	13.86 ± 4.42	660 ± 610	20.99 ± 50.6	0.00057
RANTES (ng/mL)	5.77 ± 2.22	4.19 ± 1.76	1.42 ± 0.32	0.00066
MIP-1β (pg/mL)	74.3 ± 58.6	53.4 ± 36.0	1.38 ± 0.26	0.00087
HGF (pg/mL)	532 ± 159	353 ± 160	1.65 ± 0.48	0.00098
NAP-2 (ng/mL)	184 ± 321	152 ± 280	1.28 ± 0.23	0.0011
Lymphotactin (pg/mL)	103.1 ± 95.7	64.8 ± 66.6	2.11 ± 0.99	0.0013
G-CSF (pg/mL)	65.4 ± 46.6	24.7 ± 38.2	2.65 ± 3.80	0.0016
IL-16 (pg/mL)	711 ± 573	327 ± 447	5.66 ± 6.47	0.0018
Leptin (ng/mL)	9.23 ± 12.33	1.12 ± 1.24	8.25 ± 5.07	0.0023
TARC (pg/mL)	41.4 ± 11.8	34.5 ± 11.7	1.23 ± 0.20	0.0025
MMP-1 (pg/mL)	1,170 ± 636	248 ± 301	4.72 ± 2.13	0.0045
L-Selectin (microg/mL)	1.99 ± 0.32	1.71 ± 0.20	1.16 ± 0.14	0.0049
TNFR2 (pg/mL)	434 ± 74	295 ± 104	1.63 ± 0.68	0.0051
Eotaxin 2 (pg/mL)	104 ± 49	67.7 ± 40.7	1.94 ± 1.26	0.0099

*p values were calculated using Student's paired T tests and natural log transformed data.

Values are mean ± one standard deviation.

### Comparison of heat inactivated serum, plasma, and recalified plasma with untreated serum, plasma and recalcified plasma

**Figure 3:**
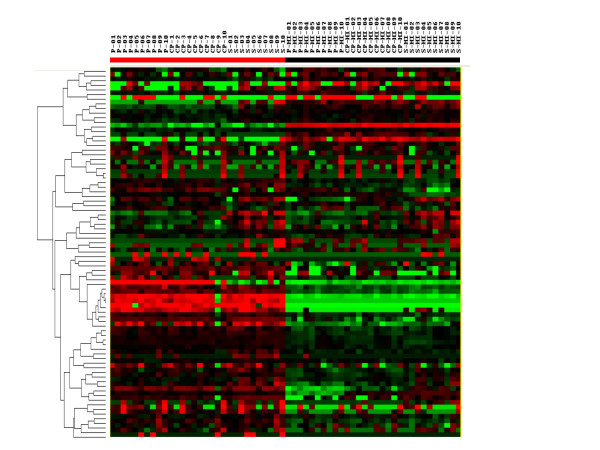
Supervised hierarchical clustering (Eisen Kendall's) of 80 soluble factors among 60 samples from 10 subjects. The samples included 10 sera (S), 10 plasma (P), 10 recalified plasma (CP), 10 heat inactivated sera (S-HI), 10 heat inactivated plasma (P-HI), and 10 heat inactivated recalified plasma (CP-HI). The serum, plasma, and recalcified plasma were prepared, stored at -80°C and tested. The heat inactivated samples were prepared, stored at -80°C, thawed, heat inactivated, stored again at -80°C and tested. Red bar encompasses regular serum, plasma and recalcified plasma. Black bar heat inactivated samples.

Since heat inactivation changed the levels of many plasma factors, stored serum, plasma, and recalified plasma samples from the 10 subjects were heat inactivated and soluble factor levels were measured. A comparison of levels of soluble factors between all heat inactivated and untreated products using supervised hierarchical clustering found that, in general, there were more differences in protein levels between heat inactivated samples and those that were not heat inactivated than among samples that were heat inactivated or among samples that were not (Figure 3). Analysis using t tests found that the levels of 19 factors differed between heat inactivated serum and heat inactivated recalcified plasma (Table 5).

**Table 5:** Comparison of the Levels of 80 Soluble Factors in Heat Inactivated Serum and Heat Inactivated Recalcified Plasma

**Factor**	**Heat Inactivated Serum**	**Heat Inactivated Recalcified Plasma**	P*
ACRP-30 (microg/mL)	15.9 ± 10.5	9.0 ± 5.8	0.000209
Amphiregulin (pg/mL)	7 ± 11	3 ± 5	N.S.
ANG-2 (pg/mL)	0 ± 0	0 ± 0	N.S.
**Apo A-1 (microg/mL)**	**89.0 ± 16.6**	**70.0 ± 17.3**	**0.000433**
**ApoB-100 (microg/mL)**	**637 ± 147**	**544 ± 98**	**0.00831**
**A-SAA (microg/mL)**	**14.7 ± 4.80**	**11.4 ± 4.12**	**0.00228**
BDNF (ng/mL)	7.23 ± 3.04	4.17 ± 1.54	N.S.
CD14 (pg/mL)	998 ± 2,439	9,922 ± 11,223	N.S.
CD40L (pg/mL)	48 ± 81	70 ± 63	N.S.
**CNTF (pg/mL)**	**0 ± 0**	**0 ± 0**	**N.S.**
**CRP (microg/mL)**	**3.52 ± 0.84**	**2.33 ± 4.71**	**N.S.**
**ENA-78 (pg/mL)**	**637 ± 392**	**343 ± 122**	**N.S.**
Eotaxin (pg/mL)	16 ± 26	25 ± 14	N.S.
Eotaxin2 (ng/mL)	1.66 ± 1.24	3.13 ± 2.33	0.000712
E-Selectin (ng/mL)	73.1 ± 16.7	70.0 ± 15.0	N.S.
**Exodus 2 (pg/mL)**	**53 ± 21**	**106 ± 55**	**0.00976**
**FGF Basic (pg/mL)**	**218 ± 175**	**160 ± 219**	**N.S.**
**Fibrinogen (ng/mL)**	**174 ± 44.2**	**217 ± 76.7**	**N.S.**
G-CSF (pg/mL)	29 ± 22	41 ± 19	N.S.
GM-CSF (pg/mL)	555 ± 267	623 ± 311	N.S.
GRO-alpha (pg/mL)	67 ± 61	36 ± 12	N.S.
**HGF (pg/mL)**	**206 ± 124**	**224 ± 43**	**N.S.**
**HGH (pg/mL)**	**164 ± 148**	**159 ± 159**	**N.S.**
**I-309 (pg/mL)**	**27 ± 64**	**21 ± 38**	**N.S.**
ICAM-1 (ng/mL)	145 ± 44.3	183 ± 48.7	0.00518
IFN-α (pg/mL)	1 ± 1.3	0.8 ± 1.3	N.S.
IFN-γ (pg/mL)	3 ± 9	3 ± 9	N.S.
**IL-11 (pg/mL)**	**0 ± 0**	**0 ± 0**	**N.S.**
**IL-12p40 (pg/mL)**	**7 ± 15**	**6 ± 10**	**N.S.**
**IL-16 (pg/mL)**	**62 ± 19**	**66 ± 21**	**N.S.**
IL-1α (pg/mL)	12 ± 26	11 ± 25	N.S.
IL-1Ra (pg/mL)	183 ± 328	150 ± 283	N.S.
IL-2 (pg/mL)	22 ± 32	21 ± 27	N.S.
**IL-2R (ng/mL)**	**1.21 ± 0.67**	**1.49 ± 0.78**	**N.S.**
**IL-2Rγ (pg/mL)**	**0 ± 0**	**0 ± 0**	**N.S.**
**IL-6 (pg/mL)**	**7 ± 13**	**7 ± 12**	**N.S.**
IL-6R (pg/mL)	30 ± 13	40 ± 10	0.00451
IL-7 (pg/mL)	5 ± 6	0.9 ± 2.6	N.S.
IL-8 (pg/mL)	7 ± 7	6 ± 9	N.S.
**IL-9 (pg/mL)**	**771 ± 1,457**	**608 ± 1,259**	**N.S.**
**IP-10 (pg/mL)**	**128 ± 77**	**141 ± 55**	**N.S.**
**ITAC (pg/mL)**	**8 ± 10**	**8 ± 6**	**N.S.**
KGF (pg/mL)	34 ± 17	26 ± 12	N.S.
LEPTIN (ng/mL)	2.15 ± 3.04	2.56 ± 3.06	N.S.
L-Selectin (microg/mL)	1.16 ± 0.32	1.06 ± 0.26	N.S.
**Lymphotactin (pg/mL)**	**94 ± 107**	**86 ± 94**	**N.S.**
**MCP-1 (pg/mL)**	**456 ± 187**	**514 ± 153**	**N.S.**
**MCP-2 (pg/mL)**	**55 ± 15**	**47 ± 18**	**N.S.**
MCP-3 (pg/mL)	0 ± 0	0 ± 0	N.S.
MCP-4 (pg/mL)	27 ± 19	30 ± 14	N.S.
MDC (pg/mL)	504 ± 127	463 ± 92	N.S.
**MIG (pg/mL)**	**113 ± 61**	**119 ± 59**	**N.S.**
**MIP-1α (pg/mL)**	**60 ± 22**	**43 ± 32**	**N.S.**
**MIP-1β (pg/mL)**	**61 ± 35**	**69 ± 37**	**N.S.**
MIP-3α (pg/mL)	6 ± 1	7 ± 2	N.S.
MIP-3β (pg/mL)	37 ± 13	56 ± 15	0.00714
MMP-1 (ng/mL)	3.17 ± 2.19	0.68 ± 0.46	0.00612
**MMP-10 (pg/mL)**	**34 ± 45**	**0 ± 0**	**N.S.**
**MMP-13 (pg/mL)**	**60 ± 114**	**0 ± 0**	**N.S.**
**MMP-2 (ng/mL)**	**205 ± 35.7**	**125 ± 31.2**	**1.09 × 10**^-05^
MMP-3 (ng/mL)	5.58 ± 2.99	1.44 ± 0.92	5.09 × 10^-07^
MMP-9 (ng/mL)	48.0 ± 35.6	15.4 ± 9.2	0.00217
MPIF-1 (pg/mL)	599 ± 184	616 ± 199	N.S.
**MPO (ng/mL)**	**45.8 ± 13.6**	**32.0 ± 6.3**	**0.00206**
**NAP-2 (microg/mL)**	**2.80 ± 2.03**	**0.21 ± 0.20**	**8.47 × 10**^-05^
**NT3 (pg/mL)**	**10 ± 9**	**5 ± 8**	**N.S.**
OPG (pg/mL)	3 ± 2	7 ± 4	0.00276
OPN (ng/mL)	2.13 ± 1.50	2.91 ± 2.68	N.S.
PAI-1 Active (pg/mL)	211 ± 179	97 ± 93	N.S.
**PAI-1 Total (ng/mL)**	**4.33 ± 2.72**	**1.67 ± 1.54**	**0.00585**
**PDGF-BB (pg/mL)**	**165 ± 171**	**85 ± 198**	**N.S.**
**RANTES (ng/mL)**	**10.8 ± 12.2**	**3.9 ± 1.5**	**N.S.**
SDF-1β (pg/mL)	173 ± 156	237 ± 172	N.S.
TARC (pg/mL)	74 ± 41	39 ± 10	N.S.
TIMP-1 (ng/mL)	121 ± 56.4	82.3 ± 17.1	N.S.
**TIMP-2 (ng/mL)**	**109 ± 25.8**	**94.0 ± 16.2**	**N.S.**
**TNF-α (pg/mL)**	**46 ± 35**	**48 ± 38**	**N.S.**
**TNF-RI (pg/mL)**	**433 ± 115**	**413 ± 111**	**N.S.**
TNF-R2 (pg/mL)	150 ± 46	584 ± 136	6.63 × 10^-07^
VCAM-1 (ng/mL)	46.5 ± 13.6	69.1 ± 12.0	0.000540

N.S. = Not Significant

*p values were calculated using Student's paired T tests and natural log transformed data.

Values are mean ± one standard deviation.

## Discussion

This study reports the protein profile of 80 factors in serum, plasma, recalcified plasma and heat inactivated components. Significant differences in soluble factor profiles and concentrations were observed among these four supplements routinely utilized in clinical cell cultures. Serum and plasma showed very distinct profiles, recalcification of plasma had little affect on the levels of most soluble factors compared to non-recalcified plasma, and heat inactivation drastically changed plasma and serum protein content

Serum differs from plasma and recalcified plasma in many ways. The levels of 18 of the 80 factors were greater in serum than in plasma. The increase in factors in serum was likely due to the stimulation of platelets by the coagulation cascade and the release of platelet factors NAP-2, RANTES, and PPGF-BB. The chemokine NAP-2 likely stimulates leukocytes leading to the production and release of cytokines, growth factors and other chemokines.

The levels of two factors were lower in serum than in plasma; fibrinogen and OPN. The lower levels of both factors are likely a direct effect of thrombin generation in serum. Thrombin catalyzes the conversion of fibrinogen to fibrin and OPN has a thrombin-cleavage site and it is degraded during the activation of the coagulation cascade [15]. The presence of thrombin-cleaved OPN in serum is likely to affect cells in culture since cleaved OPN promotes cell attachment and spreading to a greater extent than uncleaved OPN [15].

Since recalcified plasma has lower levels of fibrinogen than plasma, clots are less likely to form in media supplemented with recalcified plasma rather then plasma. However, the levels of almost all other factors are similar in plasma and recalcified plasma and the behavior of cells cultured in media plus recalcified plasma is likely be similar to those cultured in media plus plasma.

Serum or plasma is often heat inactivated to prevent the activation of complement components during cell culture [1]. We found that heat inactivation affects the levels of many of the factors studied. The levels of almost one third, 31, of the plasma factors were reduced by heat inactivation. Heating denatures proteins and causes them to become insoluble and these results show that the effects of heat inactivation are very broad.

The levels of five factors increased with heat inactivation; MPO, eotaxin 2, MCP-1, PAI-1 and TNFR1. While plasma has marked reduced levels of cellular elements compared to whole blood, it does contain some platelets and leukocytes. The increase in the levels of these 5 factor levels following heat inactivation may be the result of heat-induced cell disruption and release of intracellular contents. MPO is an abundant neutrophil secondary granule protein, PAI-1 is found in platelets [16], TNFR1 is found on all leukocytes, eotaxin 2 in monocytes and macrophages [17] and MCP-1 in monocytes and T cells. Since the levels of many chemokines, growth factors and cell adhesion molecules changed as a result of heat inactivation, the behavior of cells in media supplemented with heat inactivated plasma is likely to be much different than the behavior of cells in media plus plasma.

Our laboratory often uses heat inactivated recalcified plasma rather then heat inactivated serum. Our multiplex protein analysis revealed several differences between the two types of supplements. Since the levels of many chemokines and cell adhesion molecules differed in heat inactivated serum and heat inactivated recalified plasma, the behavior of cells cultured in media plus heat inactivated serum may be different than from those cultured in media plus heat inactivated recalcified plasma. These differences do not preclude the use of heat inactivated recalcified plasma, but when it is used in place of heat inactivated serum the cells must be carefully analyzed to ensure the final product has not changed.

Media without serum or plasma supplements have been used to culture a number of cell types including hematopoietic progenitor cells, dendritic, neutrophils, and leukemia cells [2,4,9,10,18,19]. However, when media with serum or plasma must be used, our report provides a useful characterization of serum/plasma components which can aid in selecting the most appropriate blood supplement for a particular type of cell culture or clinical hematological procedure. Additionally it can shed light on the soluble factors that are missing when investigators subject cultures to serum free media conditions

## Supplementary Material

Additional File 1 Table comparing the levels of 80 soluble factors among serum, plasma, recalcified plasma, and heat inactivated plasma. This table provides the levels of soluble factors in the 40 serum, plasma, recalcified plasma, and heat inactivated plasma shown in figure 1. Click here for file
