# Supplementary material for: Comparison of proteomic profiles of serum, plasma, and modified media supplements used for cell culture and expansion
Source: J Transl Med. 2006 Oct 4;4:40. doi: 10.1186/1479-5876-4-40 (PMC1601968; doi:10.1186/1479-5876-4-40)
Supplement: Additional File 1 — Table comparing the levels of 80 soluble factors among serum, plasma, recalcified plasma, and heat inactivated plasma. This table provides the levels of soluble factors in the 40 serum, plasma, recalcified plasma, and heat inactivated plasma shown in figure 1. [file 1479-5876-4-40-S1.doc]

**Appendix 1. Comparison of the Levels of 80 Soluble Factors Among Serum, Plasma, Recalcified Plasma, and Heat Inactivated Plasma**

|  | **Serum** | **Plasma** | **Recalcified Plasma** | **Heat Inactivated Plasma** |
| --- | --- | --- | --- | --- |
| ACRP-30 (microg/mL) | 15.5 ± 14.8 | 11.2 ± 11.3 | 9.4 ± 5.0 | 7.83 ± 4.41 |
| Amphiregulin (pg/mL) | 44 ± 66 | 7± 9 | 3 ± 6 | 7.3 ± 16 |
| ANG-2 (pg/mL) | 240 ± 129 | 212 ± 95 | 197 ± 109 | 0 ± 0 |
| **Apo A-1 (microg/mL)** | **208 ±38.5** | **173± 46.9** | **174 ± 36.1** | **162 ± 29.1** |
| **ApoB-100 (microg/mL)** | **535± 21** | **433 ± 16** | **358± 12** | **338 ± 14** |
| **A-SAA (microg/mL)** | **39.5 ± 19.8** | **30.3 ± 11.1** | **30.2 ± 9.91** | **34.3 ± 10.8** |
| BDNF (ng/mL) | 8.43 ± 1.54 | 8.53 ± 4.04 | 7.81 ± 2.94 | 11.99 ± 3.94 |
| CD14 (ng/mL) | 1.82 ± 5.74 | 3.82 ± 7.21 | 2.68 ± 6.35 | 5.80 ± 6.13 |
| CD40L (pg/mL) | 240± 311 | 188 ± 278 | 172 ± 282 | 112 ± 107 |
| **CNTF (ng/mL)** | **1.06± 1.01** | **1.07 ± 1.10** | **1.39 ± 1.32** | **1.82 ± 1.36** |
| **CRP(microg/mL)** | **1.82 ± 1.60** | **1.75 ± 1.73** | **1.75 ± 1.72** | **0.73 ± 0.92** |
| **ENA-78 (pg/mL)** | **1,449 ± 787** | **770 ± 403** | **719 ± 317** | **955 ± 592** |
| Eotaxin (pg/mL) | 61 ± 59 | 105 ± 49 | 108 ± 48 | 68 ± 41 |
| Eotaxin2 (ng/mL) | 1.98 ± 1.37 | 0.99 ± 0.58 | 1.04 ± 0.747 | 2.35 ±1.38 |
| E-Selectin (ng/mL) | 46.0 ± 12.1 | 406 ± 11.3 | 38.1 ± 11.1 | 33.9 ± 13.4 |
| **Exodus 2 (pg/mL)** | **57 ± 17** | **49 ± 11** | **50 ± 15** | **52 ± 18** |
| **FGF Basic (pg/mL)** | **292 ± 320** | **87 ± 151** | **111 ± 144** | **124 ±181** |
| **Fibrinogen (microg/mL)** | **3.13± 7.21** | **4,812 ± 1,780** | **262 ± 383** | **0.10 ± 0.30** |
| G-CSF (pg/mL) | 55 ± 67 | 65 ± 47 | 43 ± 44 | 25 ± 38 |
| GM-CSF (pg/mL) | 179 ± 559 | 163 ± 402 | 180 ± 412 | 291 ± 502 |
| GRO-α (pg/mL) | 30 ± 30 | 11 ± 7 | 11 ± 4 | 10 ± 4 |
| **HGF(pg/mL)** | **1,043 ± 650** | **532 ± 159** | **517 ± 177** | **353 ± 160** |
| **HGH (pg/mL)** | **92 ± 84** | **80 ± 88** | **79 ± 71** | **88 ± 103** |
| **I-309 (pg/mL)** | **6 ± 15** | **8 ± 15** | **6 ± 14** | **10 ± 20** |
| ICAM-1 (ng/mL) | 355 ± 44.8 | 321 ± 37.9 | 320 ± 42.1 | 201 ± 24.2 |
| IFN-α (pg/mL) | 7 ± 7 | 11 ± 11 | 4 ± 5 | 5 ± 6 |
| IFN-γ (pg/mL) | 1 ± 4 | 3 ± 5 | 2 ± 4 | 1 ± 2 |
| **IL-11 (pg/mL)** | **1 ± 2** | **8 ± 11** | **8 ± 10** | **57 ± 59** |
| **IL-12p40 (pg/mL)** | **36 ± 54** | **34 ± 48** | **28 ± 42** | **33 ± 51** |
| **IL-16 (pg/mL)** | **493 ± 261** | **711 ± 573** | **465 ± 513** | **327 ± 447** |
| IL-1α (pg/mL) | 11 ± 27 | 10 ± 19 | 8 ± 22 | 8 ± 17 |
| IL-1Ra (pg/mL) | 252 ± 515 | 208± 392 | 163 ± 354 | 136 ± 285 |
| IL-2 (pg/mL) | 17 ± 26 | 18± 20 | 17 ± 21 | 19 ± 25 |
| **IL-2R (pg/mL)** | **1,003 ± 562** | **859 ± 378** | **860 ± 467** | **857 ± 553** |
| **IL-2Rγ (pg/mL)** | **22 ± 41** | **24 ± 62** | **63 ± 200** | **0.0 ± 0** |
| **IL-6 (pg/mL)** | **4 ± 9** | **4 ± 8** | **3 ± 7** | **3 ± 7** |
| IL-6R (ng/mL) | 14.6 ± 4.96 | 13.2 ± 4.4 | 11.4 ± 5.5 | 0.04 ± 0.07 |
| IL-7 (pg/mL) | 11 ± 19 | 8 ± 14 | 7 ± 14 | 7 ± 12 |
| IL-8 (pg/mL) | 10 ± 14 | 9 ± 11 | 10 ± 16 | 7 ± 11 |
| **IL-9 (pg/mL)** | **45 ± 80** | **62 ± 133** | **55 ± 78** | **93 ± 141** |
| **IP-10 (pg/mL)** | **77 ± 92** | **76 ± 73** | **71 ± 58** | **92 ± 70** |
| **ITAC (pg/mL)** | **78 ± 16** | **51 ± 7** | **48 ± 18** | **18 ± 10** |
| KGF (pg/mL) | 43 ± 60 | 2 ± 5 | 6 ± 12 | 0 ± 0 |
| LEPTIN (ng/mL) | 10.2 ± 15.0 | 9.23 ± 12.3 | 9.78 ± 14.2 | 1.12 ± 1.24 |
| L-Selectin (microg/mL) | 2.24 ± 0.35 | 1.99 ± 0.31 | 1.99 ± 0.19 | 1.71 ± 0.20 |
| **Lymphotactin (pg/mL)** | **125 ± 104** | **103 ± 96** | **77 ± 51** | **65 ± 67** |
| **MCP-1 (pg/mL)** | **303 ± 141** | **361± 97** | **359 ± 82** | **425 ± 111** |
| **MCP-2 (pg/mL)** | **47 ± 11** | **31 ± 10** | **28 ± 10** | **29 ± 12** |
| MCP-3 (pg/mL) | 5 ± 4 | 2 ± 2 | 0.5 ± 1.5 | 0 ± 0 |
| MCP-4 (pg/mL) | 29 ± 19 | 23 ± 10 | 22 ± 9 | 20 ± 10 |
| MDC (pg/mL) | 341± 56 | 350 ± 71 | 271 ± 50 | 220 ± 36 |
| **MIG (pg/mL)** | **469 ± 449** | **421 ± 363** | **355 ± 238** | **444 ± 255** |
| **MIP-1α (pg/mL)** | **96 ± 22** | **56 ± 9** | **54 ± 23** | **0 ± 0** |
| **MIP-1β (pg/mL)** | **99 ± 64** | **74 ± 59** | **60 ± 25** | **53 ± 36** |
| MIP-3α (pg/mL) | 17 ± 7 | 12 ± 6 | 9 ± 3 | 2 ± 2 |
| MIP-3β (pg/mL) | 107 ± 37 | 88 ± 22 | 83 ± 28 | 44 ± 12 |
| MMP-1 (ng/mL) | 2.63 ± 1.90 | 1.17 ± 0. 637 | 1.02 ± 0.59 | 0.25 ± 0.30 |
| **MMP-10 (pg/mL)** | **245 ± 224** | **292± 183** | **273 ± 242** | **3 ± 10** |
| **MMP-13 (pg/mL)** | **160 ± 280** | **66 ± 125** | **113 ± 229** | **0 ± 0** |
| **MMP-2 (ng/mL)** | **117 ± 20.1** | **114 ± 18.4** | **106 ± 16.9** | **34.1 ± 21.4** |
| MMP-3 (ng/mL) | 15.8 ± 5.8 | 13.9 ± 4.4 | 13.9 ± 6.8 | 0.66 ± 0.610 |
| MMP-9 (ng/mL) | 32.1 ± 23.5 | 11.4 ± 4.42 | 20.8 ± 32.2 | 9.76 ± 6.58 |
| MPIF-1 (ng/mL) | 1.92 ± 0.46 | 1.62 ± 0.37 | 1.45 ± 0.52 | 0.48 ± 0.21 |
| **MPO (pg/mL)** | **257 ± 249** | **359 ± 245** | **348 ± 670** | **2,703 ± 1,171** |
| **NAP-2 (microg/mL)** | **4.79 ± 7.16** | **0.18 ± 0.32** | **0.26 ± 0.31** | **0.15 ± 0.28** |
| **NT3 (pg/mL)** | **25 ± 47** | **0 ± 0** | **0 ± 0** | **1 ± 3** |
| OPG (pg/mL) | 0 ± 0 | 0.9 ± 1.2 | 1.5 ± 1.7 | 1 ± 1 |
| OPN (ng/mL) | 1.82 ± 1.18 | 4.12 ± 1.48 | 3.92 ± 1.38 | 2.90 ± 2.34 |
| PAI-1 Active (ng/mL) | 5.82 ± 4.61 | 4.19 ± 4.60 | 3.81± 3.75 | 0.07 ± 0.09 |
| **PAI-1 Total (ng/mL)** | **4.18 ± 3.17** | **0.93 ± 0.75** | **1.12± 1.03** | **1.71 ± 1.83** |
| **PDGF-BB (pg/mL)** | **214 ± 225** | **33 ± 20** | **24 ± 22** | **7 ± 18** |
| **RANTES (ng/mL)** | **22.0 ± 20.2** | **5.8 ± 2.2** | **7.1± 2.4** | **4.2 ± 1.9** |
| SDF-1beta (pg/mL) | 710 ± 218 | 798 ± 137 | 630 ± 176 | 336 ± 96 |
| TARC (pg/mL) | 110 ± 78 | 41 ± 12 | 42 ± 12 | 34 ± 12 |
| TIMP-1(ng/mL) | 106 ± 25.2 | 71.8 ± 8.4 | 71.4 ± 13.6 | 71.8 ± 14.2 |
| **TIMP-2 (ng/mL)** | **157 ± 31.5** | **137± 21.0** | **142 ± 25.8** | **132 ± 25.1** |
| **TNF-α (pg/mL)** | **51 ± 36** | **62 ± 42** | **55 ± 40** | **75 ± 49** |
| **TNF-RI (pg/mL)** | **582 ± 139** | **422 ± 103** | **485 ± 94** | **499 ± 116** |
| TNF-R2 (pg/mL) | 491 ± 90 | 434 ± 74 | 435 ± 144 | 295 ± 104 |
| VCAM-1 (ng/mL) | 598 ± 158 | 556 ± 133 | 496 ± 184 | 113 ± 43.4 |
